# TranscriptDB: a transcript-centric database to study eukaryotic transcript conservation and evolution

**DOI:** 10.1093/nar/gkae995

**Published:** 2024-11-12

**Authors:** Wend Yam D D Ouedraogo, Aida Ouangraoua

**Affiliations:** Department of Computer Science, Faculté des sciences, Université de Sherbrooke, 2500 Boulevard de l'Université, Sherbrooke, QC J1K 2R1, Canada; Department of Computer Science, Faculté des sciences, Université de Sherbrooke, 2500 Boulevard de l'Université, Sherbrooke, QC J1K 2R1, Canada

## Abstract

Eukaryotic genes can encode multiple distinct transcripts through the alternative splicing (AS) of genes. Interest in the AS mechanism and its evolution across different species has stimulated numerous studies, leading to several databases that provide information on AS and transcriptome data across multiple eukaryotic species. However, existing resources do not offer information on transcript conservation and evolution between genes of multiple species. Similarly to genes, identifying conserved transcripts—those from homologous genes that have retained a similar exon composition—is useful for determining transcript homology relationships, studying transcript functions and reconstructing transcript phylogenies. To address this gap, we have developed TranscriptDB, a database dedicated to studying the conservation and evolution of transcripts within gene families. TranscriptDB offers an extensive catalog of conserved transcripts and phylogenies for 317 annotated eukaryotic species, sourced from Ensembl database version 111. It serves multiple purposes, including the exploration of gene and transcript evolution. Users can access TranscriptDB through various browsing and querying tools, including a user-friendly web interface. The incorporated web servers enable users to retrieve information on transcript evolution using their own data as input. Additionally, a REST application programming interface is available for programmatic data retrieval. A data directory is also available for bulk downloads. TranscriptDB and its resources are freely accessible at https://transcriptdb.cobius.usherbrooke.ca.

## Graphical Abstract

**Graphical Abstract:**
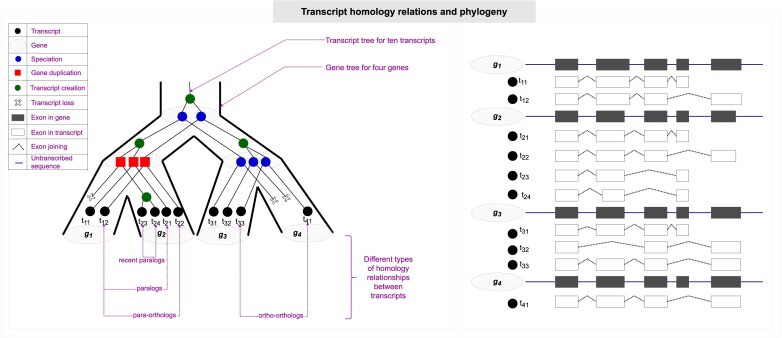

## Introduction

Since the discovery of exons and introns in 1977 (1), there has been a growing interest in the alternative processing of eukaryote genes, which significantly contributes to the diversity of the eukaryote transcriptome (2). In particular, through alternative splicing (AS), the splicing of different combinations of introns makes it possible to produce different transcripts from a gene (3). It is now widely acknowledged that more than half of human genes undergo AS, with studies indicating an even higher prevalence in the eukaryotic kingdom (4,5) and evolutionary conserved impacts on the proteome (6). The dysregulation of the splicing machinery has also been linked to both rare and complex diseases (7–12). With the growth of RNA sequencing (RNA-seq) data and the large amount of publicly available transcriptome data, a major challenge is to elucidate the functional relevance of transcripts (4,13). Many databases have been designed to study AS and transcript diversity, such as H-DBAS (14) and ASTD (15,16). However, existing databases fail to provide information about transcript conservation and evolution between genes across multiple species.

Understanding transcript evolution is important for better understanding the relationship between gene evolution and the alternative processing of genes (5,17–20), and the evolution of gene expression (21–23). Interest in homology relationships between genes has led to the development of methods and databases primarily aimed at identifying orthology and paralogy relations between homologous genes (24–29). Similarly, knowing more about homology relationships between transcripts has the potential to enhance gene and transcript annotation transfer between related species (17,28). Identifying conserved transcripts with similar exon compositions between genes helps to identify conserved functions across multiple genes and species (20). Studying conserved transcripts and the evolution of transcript sets produced by homologous genes is thus crucial for enhancing our understanding of AS regulation and aiding in assessing the support value and function of predicted transcripts. In particular, reconstructing transcript phylogenies can contribute to improving transcript annotation by aiding in reconstructing spliced transcripts from RNA-seq data without a reference genome, by using the transcriptome of related genomes (17,18,20). We have developed computational methods for inferring orthology and paralogy relations at the transcript level (19) and for reconstructing transcript phylogenies based on transcript homology relations (20).

Here, we present TranscriptDB, a transcript-centric database designed for the study of transcript conservation and evolution within gene trees. The computed data stored in TranscriptDB rely on two computational methods developed to infer orthology and paralogy relations between transcripts (19) and to reconstruct transcript phylogenies (20). The database offers a comprehensive catalog of data on transcript homology relation types and transcript phylogenies within Ensembl-annotated gene trees (30). In addition to contributing to elucidating the functional relevance of transcripts and enhancing gene and transcript annotation, TranscriptDB will aid in the development of accurate methods for identifying transcript conservation across genes and species, as well as for the inference of transcript families and their phylogenies. Access to TranscriptDB does not necessitate user registration or login, and the database is freely available. Users can access the database and download data via the web browser interface, perform bulk downloads from the data directory or use the REST application programming interface (API) provided with the database.

## Materials and methods

### Gene and transcript data from the Ensembl Compara v111 database

The current gene and transcript data were obtained from the Ensembl Compara v111 database (30), providing information that includes gene identifiers, approved gene names, gene sequences, gene locations (chromosomal positions, strands, genome identifiers and chromosome identifiers), transcript identifiers, transcript support levels, transcript exon compositions, exon locations, transcript coding sequence indicated by the chromosomal positions of start and stop codons, gene homology relations and gene trees. Each gene tree represents the evolutionary relationships within a gene family composed of a set of homologous genes. The Gene Ontology terms (31) associated with the transcripts of each retrieved gene are also provided, when available.

### Computed data on transcript homology relations and transcript phylogenies

For each gene, we computed its transcribed sequence. The transcribed sequence of a gene is defined as the concatenation of all exon sequences that are included in at least one transcript produced from the gene. Next, we performed a multiple alignment of all transcribed sequences from a gene family. To account for the amino acid level in our alignment, we used MACSE (32). In Ensembl version 111, the maximum number of genes included in a gene family is 1500. The alignment process is particularly time-consuming for families with a high number of genes. To address this limitation, we used MACSE for gene families consisting of 100 genes or fewer (40 671 families out of 54 673) and employed Kalign (33) for larger families. Once the alignment of all transcribed sequences was obtained for a gene family, we mapped each transcript sequence of the family to the alignment to infer the multiple sequence alignment of transcripts.

From the multiple sequence alignment of transcripts, we used a computational method based on a reciprocal best-hit approach and gene-level homology relationships to infer different types of homology relations between transcripts (19). The method outputs clusters of orthologous transcripts. Two types of homology relationships between transcripts are identified within these clusters:

- Recent paralogs are identified as two transcripts from the same gene that are more similar to each other—in terms of the exon structure—than to any other transcript in the gene family.
- Orthologs are identified as two transcripts from two different genes that are more similar to each other—in terms of the exon structure—than to any other transcript in the gene family. Ortho-orthologs are orthologous transcripts from orthologous genes, while para-orthologs are orthologous transcripts from paralogous genes.

By definition (19), two transcripts are paralogs if they descend from two distinct ancestral transcripts that were in the same ancestral gene at some point of the evolution, otherwise the two transcripts are orthologs. Note that this definition of orthologous transcripts differs from the definition used in previous studies [for instance (34)] where orthologous transcripts (also known as splicing orthologs) are identified as both having a similar structure and belonging to orthologous genes. Here, we refer to such transcripts as ortho-orthologs.

**Figure 1.**
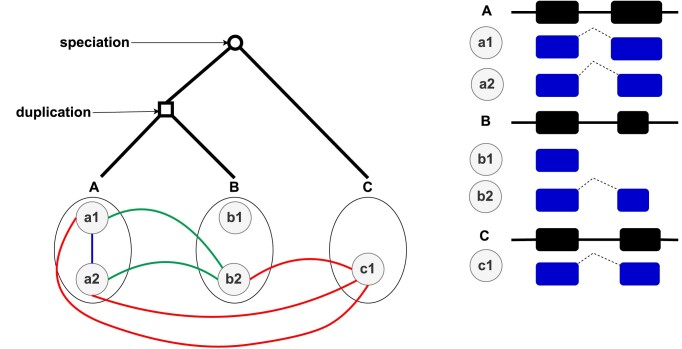
Transcript homology relation types. Illustration of a gene tree representing the evolutionary history of three genes: A, B and C. In the tree, square internal nodes represent duplications, while circular internal nodes represent speciations. A and B are paralogs as their least common ancestor in the tree is a duplication, while A and C are orthologs as their least common ancestor is a speciation. Their respective transcripts, denoted as $\lbrace \texttt {a1}, \texttt {a2}\rbrace$, $\lbrace \texttt {b1}, \texttt {b2}\rbrace$ and $\lbrace \texttt {c1}\rbrace$, are depicted as nodes beneath them. The transcribed sequences of the three genes are indicated by rectangles following the name of each gene on the right side of the figure, where solid lines represent introns or untranslated regions, and dashed lines indicate the concatenation of exons at the transcript level. The different types of homology relations between transcripts are represented by colored edges: a blue edge signifies a recent-paralogy relation, a green edge represents a para-orthology and a red edge indicates an ortho-orthology. For instance, the transcripts a1 and a2 are recent paralogs. The connected component that includes the transcripts a1, a2, b2 and c1 represents a cluster of orthologous transcripts.

The prefix in these terms—ortho-orthologs and para-orthologs—refers to the homology at the gene level, while the suffix is used to describe the transcript-level homology. Figure 1 provides an illustration of the different types of homology relationships between transcripts that can be computed.

Once the clusters of orthologous transcripts are computed, and the types of homology relations are inferred, we compute transcript phylogenies using a computational method described in (20). This method leverages the information of transcript orthology within the clusters to reconstruct transcript phylogenies—transcript forests—in congruence with the corresponding gene trees. In the first step of the method, a dendrogram is constructed to indicate how clusters—represented as orthologous transcript subtrees—should be merged to reconstruct a transcript phylogeny. Based on the phylogenetic distance defined in (20), if two clusters are too distant, the method does not merge them and considers that they belong to distinct transcript trees, and thus distinct transcript families within the same gene tree. In this case, the two corresponding sets of transcripts are called analogs.

A transcript family is then defined as a subset of transcripts derived from genes within the same gene family, for which we are able to infer a single transcript tree.

**Figure 2.**
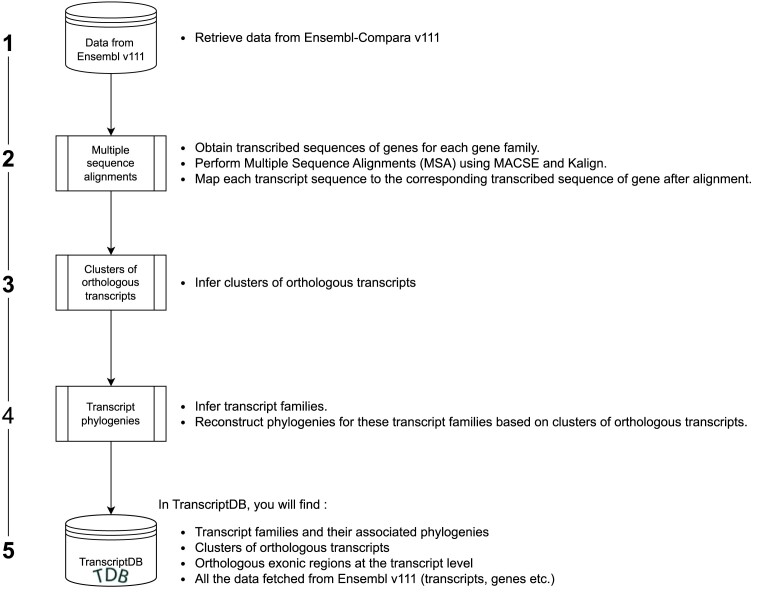
The TranscriptDB pipeline. The data stored in TranscriptDB are the result of five main steps. Steps 2 and 3 correspond to the methods described in (19) and (20), respectively.

Figure 2 shows the step-by-step procedure to generate the computed data stored in TranscriptDB.

### Database organization

The information on genes, gene trees, transcripts and transcript families is stored in TranscriptDB within four main tables in a relational database created using PostgreSQL version 15.6. Since a gene can encode multiple transcripts, the Genes table and Transcripts table are related with a one-to-many cardinality. A gene tree represents the evolution of a set of homologous genes, and a transcript family represents a set of homologous transcripts. Therefore, the cardinality between the Genetrees table and the Genes table is one-to-many. Similarly, the cardinality between the TranscriptFamilies table and the Transcripts table is one-to-many. Additionally, the TranscriptFamilies table maintains a many-to-one cardinality with the Genetrees table.

**Figure 3.**
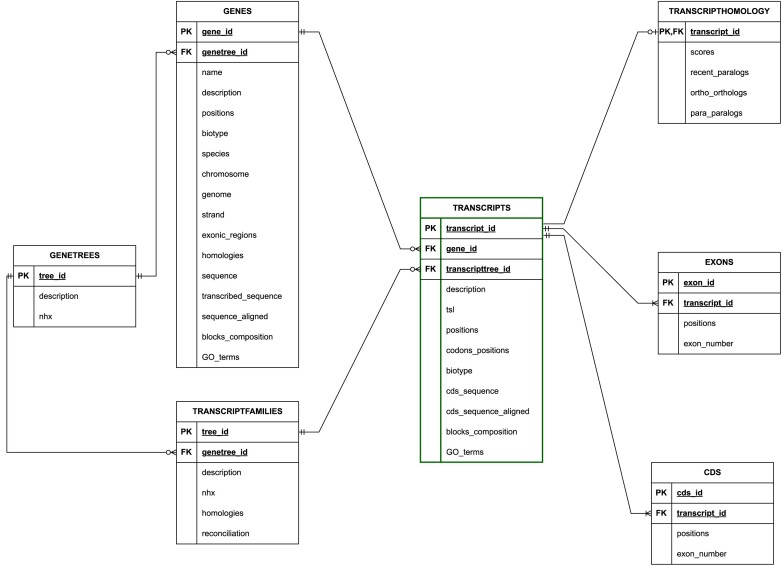
The entity relationship diagram of TranscriptDB.

Moreover, there are three child tables: the Exons table, the CDS table and the Transcripthomology table. The first two are associated with the Transcripts table in a one-to-many cardinality, while the last one is connected with a one-to-one cardinality to the Transcripts table. The database organization is illustrated in the entity relationship diagram provided in Figure 3.

### Web interface and API implementation

The web interface was built using the Next.js 14.1.4 framework, complemented with various client-side languages such as HTML, Tailwind CSS and JavaScript, as well as React libraries. TranscriptDB was implemented by deploying a Linux-based Apache 2.4.41 web server on a Ubuntu 20.04.6 LTS system. The web interface is designed to be responsive, allowing access from screens of different sizes, including phones, tablets and computers. The web interface, accessible at https://transcriptdb.cobius.usherbrooke.ca, offers a user-friendly and comprehensive experience. In addition, a RESTful API built using Next.js 14.1.4 is provided, to facilitate the communication between users and the database, and programmatical access to the database. Users can retrieve JSON-formatted data using the GET method without requiring authentication. A complete tutorial on how to use the web interface is available at https://transcriptdb.cobius.usherbrooke.ca/help. The complete API documentation is also available at https://transcriptdb.cobius.usherbrooke.ca/api-docs. We also offer web access to a structured repository where all the computed data of the database are stored. Users can access this repository for bulk downloads at https://transcriptdb.cobius.usherbrooke.ca/data.

## Results

### Database content and current statistics

TranscriptDB stores information on 12 949 613 transcripts derived from 6 700 560 genes across 317 eukaryote species. The start and end locations of exons for each transcript are also stored, totaling 150 435 355 exons, along with information indicating whether they entirely consist of coding sequence or contain untranslated regions. Furthermore, the database contains 14 056 multiple sequence alignments computed using KAlign and 40 617 computed using MACSE. A total of 80 733 transcript trees (transcript families) out of 54 673 gene trees (gene families) were computed and stored in TranscriptDB. As some genes are not grouped into gene families in the Ensembl database, not all transcripts in TranscriptDB are members of a transcript family. The information for these transcripts remains available, as does the information for the corresponding genes, even though they are not grouped into gene families. TranscriptDB contains, for each transcript family, the pairwise homology relationships between transcripts and the corresponding labeled transcript tree reconciled with the corresponding gene tree.

### Web interface usage

The web interface primarily allows users to browse and explore information about transcripts, as well as computed information regarding their homology relationships and phylogenies. Three modules are provided to users in order to

- search and explore information related to transcripts, transcript families, genes and gene families, and visualize and download the available data;
- retrieve clusters of orthologous transcripts given user-input data, which provides insights into evolutionary relationships and functional similarities between transcripts, and obtain user-friendly displays of the results;
- retrieve transcript phylogenies given user-input data, visualize the results in a user-friendly display and download the results for further analysis or reference.

The web interface offers help documentation and tutorials to get started with the web interface and the REST API.

### Exploring, searching and browsing: a user case

On the main page, a search engine enables users to retrieve information from TranscriptDB by entering specific keywords within designated categories. This feature facilitates quick and customized searches within the database. The categories that can be selected are Ensembl Transcript ID, TDB Transcript Family ID, Ensembl Gene ID, Ensembl Gene Tree ID, Gene Name, Taxon and Common Species Name. For example, a user can perform a search for an Ensembl transcript ID by selecting the category Ensembl Transcript ID and then entering the transcript ID ENSDCDT00000063049.

Beneath the search engine, an example keyword is provided for each category, offering users references on how to structure their queries within the respective categories. By default, the current version of data stored is from Ensembl version 111. Once the query is executed, users are directed to the response data page, which provides a comprehensive visualization of the retrieved information. This page also offers functionality to redirect the query to the Ensembl browser or to navigate between different pages within TranscriptDB.

**Figure 4.**
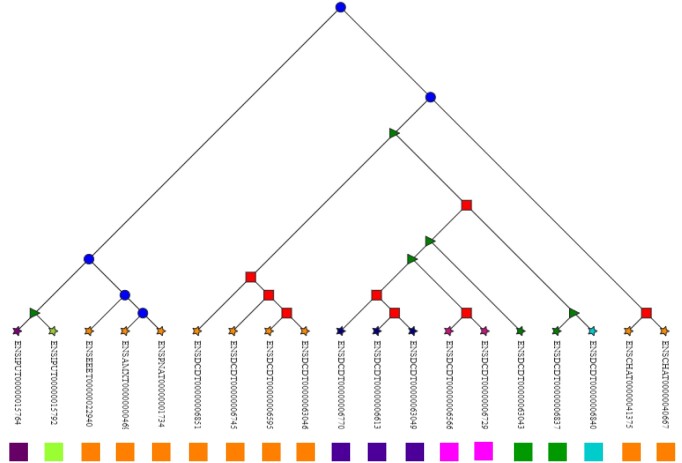
View of transcript conservation in a transcript tree. The transcript tree identified in TranscriptDB as TDBTT00990000204364∣.2, derived from the Ensembl gene tree ID ENSGT00990000204364. Clusters of orthologous transcripts at leaves are depicted as colored rectangles. Circular internal nodes represent speciation events, square internal nodes are gene duplications and triangular nodes are transcript creation events.

- *Transcript search*. The response page will present three primary sections. The initial section consists of a dynamically generated table summarizing key details about the queried transcript, including the start and end locations in the chromosome, the corresponding gene and the corresponding transcript tree, if applicable. The second section provides an overview of the exon composition of the transcript sequence. Users can interact with each exon segment to access details such as the exon identifier and its sequence. Furthermore, users can download the sequence of a specific exon, the sequence of the transcript and the exon–intron composition for further use. In the third section, users can gain a deeper understanding of the splicing structure of transcripts through the visualization of the CDS table. This table highlights the exons that are translated and provides their locations within the transcript sequence. The button *homologs*, when clicked, gives access to the alignment of the transcript within the transcript family, along with the sequence of the exonic regions conserved within the family and associated similarity score with each transcript included in the transcript family. Users can filter the homologs table based on different homologous relationships. As the homologs table is modified, an updated alignment of the selected transcripts is displayed, with the ability to highlight relevant transcripts. The corresponding cluster of orthologous transcripts that includes the transcript queried is also displayed. An illustration of the transcript tree reconstructed that highlights the clusters of orthologous transcripts can be accessed by visiting the TDB Transcript Family ID page, as shown in Figure 4. On this page, users will have access to comprehensive information about the transcript family.
- *Gene search*. To search for an annotated Ensembl gene ID in TranscriptDB, users can select the category Ensembl Gene ID and then enter the gene ID of interest. Similarly to querying a transcript, the response page is segmented into three primary sections. The first section provides a description of the gene specified in the input, while the second section offers an overview of the gene’s structure and model. In the third section, a comprehensive table describes all the transcripts associated with the gene. Additionally, users have the option to download the transcript table, as well as the gene model and structure, providing access to the sequence of each transcript. Clicking the button *homologs* grants users access to the homologs section, where they can apply filters to the homologs table for orthologs (1:1, 1:many, many:many) or paralogs (in-paralogs and out-paralogs). Upon modifying the homologs table, a dynamic gene tree is presented, where the selected genes are highlighted in yellow. When searching for a Gene Name, such as TAF6, a general description is provided and a table of transcripts will be displayed, providing descriptions of all transcripts derived from that specific gene name across species. Users can apply filters within the table to retrieve the desired information. Additionally, a summarized histogram showcasing the number of transcripts per species is provided. Users can quickly identify the genome that possesses the highest number of transcripts, for example.
- *Gene family search*. To search for an annotated Ensembl gene tree ID in TranscriptDB, users can choose the category Ensembl Gene Tree ID and enter the specific ID of interest. For instance, by entering the Ensembl gene tree ID ENSGT00990000204364 into the search field, users will be presented with relevant information about the gene tree. This includes details such as the number of transcript families derived from the gene tree, the number of speciation nodes, duplication nodes and leaves associated with the gene tree. The response page places emphasis on the transcripts derived from all the genes that composed the gene tree. In one section, a table describes these transcripts, while another section focuses on the gene tree, highlighting both duplication and speciation nodes to help in understanding the evolutionary relationships.
- *Taxon or common species name search*. Users have the option to search for all the transcripts in a specific eukaryote species in TranscriptDB by selecting the Common Species Name category if they wish to enter the common name of the species. Alternatively, they can choose the Taxon category and input the scientific name of the species. For example, they can enter saccharomyces_cerevisiae or *Saccharomyces cerevisiae* for the respective options of Taxon or Common Species Name. Users can find the names of species on the homepage, where a structured table displays all of them. Upon receiving the query, the response page is divided into two sections. The first section offers an overview of the transcript prevalence in relation to genes, providing information on the number of transcripts within the corresponding species and the count of gene and transcript families in which, respectively, genes and transcripts from this species are found. The second section presents a table focused on transcripts, displaying details such as the associated gene name, transcript family, if applicable, and protein description.

All tools available in TranscriptDB can be accessed via the Tools page. Users can compute clusters of orthologous transcripts or reconstruct transcript phylogenies to infer homologies between a set of transcripts. Compared to existing comparable tools such as the ASES web server (35), which provides an interactive platform to explore the contributions of AS to transcript diversity in evolution, the TranscriptDB web server offers greater flexibility by allowing users to input their own data.

### Database access and data download

TranscriptDB is accessible at https://transcriptdb.cobius.usherbrooke.ca. It is freely accessible to the public without any restrictions, allowing anonymous users to explore its data and tools. The tools used to compute the data are also publicly accessible, and their source codes are open source (19,20). The TranscriptDB API is also publicly accessible. Help documentation and tutorials are provided to get started with the TranscriptDB web interface and the TranscriptDB API. A structured repository is also available for bulk downloads of data (see the ‘Data availability’ section for details). Users can download data tables, figures and sequences at their convenience.

## Applications and discussion

TranscriptDB provides an interactive web interface for studying the evolution of eukaryotic genes in the context of AS, with a focus on highlighting the evolution of transcripts and exons. Detailed information on transcript exon composition is stored in TranscriptDB, serving as a dataset for methods aimed at inferring clusters of orthologous transcripts based on exon composition comparisons. TranscriptDB provides information on clusters of orthologous transcripts identified across a wide range of species, offering added value over state-of-the-art databases, which typically provide information on orthologous transcripts between only two or three species (34). This dataset will also be useful for evaluating future methods to compute orthology relations between transcripts, by offering established clusters of orthologous transcripts to be used as benchmark data.

TranscriptDB enriches the study of conserved transcripts by providing rapid access to transcript conservation data through its interface. It includes graphical representations that illustrate conserved transcripts within their respective transcript trees. The availability of conserved transcript data is particularly beneficial for studying the evolutionary relationships of these transcripts and exploring different types of homology among them. TranscriptDB is dedicated to investigating data to gain insights into the impact of AS on gene and transcript evolution.

Each transcript family archived in TranscriptDB represents a comprehensive dataset that supports the development of methods for reconstructing the evolutionary histories of transcripts. TranscriptDB serves as a resource for annotating functional proteins across genomes and facilitating the discovery of homology relationships between transcripts spanning multiple genes and species.

## Conclusion

TranscriptDB is a new database developed to study the conservation of transcripts and their evolution within gene tree. The data stored are public and accessible through a web interface. The web interface allows users to query the database and execute its tools. To the best of our knowledge, TranscriptDB is currently the only existing database focused on transcript conservation and evolution. Future work will involve integrating more data from various versions of the Ensembl Compara database and improving the method for inferring orthologous transcripts by accounting for the structure of corresponding proteins. This will contribute to enhancing our understanding of gene, transcript and splicing evolution.

## Data Availability

TranscriptDB is freely accessible to users at https://transcriptdb.cobius.usherbrooke.ca; web interface tutorial: https://transcriptdb.cobius.usherbrooke.ca/help; REST API documentation: https://transcriptdb.cobius.usherbrooke.ca/api-docs; bulk downloads: https://transcriptdb.cobius.usherbrooke.ca/data; web server tools: https://transcriptdb.cobius.usherbrooke.ca/tools.
